# Zeolitic Imidazole Framework-90-Based Pesticide Smart-Delivery System with Enhanced Antimicrobial Performance

**DOI:** 10.3390/nano12203622

**Published:** 2022-10-15

**Authors:** You Liang, Sijin Wang, Hongqiang Dong, Siwen Yu, Huijuan Jia, Jin Wang, Yijia Yao, Yuanfeng Wang, Jiehui Song, Zhongyang Huo

**Affiliations:** 1 Co-Innovation Center for Modern Production Technology of Grain Crop/Jiangsu Key Laboratory of Crop Genetics and Physiology, Yangzhou University, Yangzhou 225009, China; 2 Xinjiang Production and Construction Corps Key Laboratory of Protection and Utilization of Biological Resources in Tarim Basin, Tarim University, Alaer 843300, China

**Keywords:** ZIF-90, kasugamycin, pH-responsive, pesticide delivery, *Magnaporthe oryzae*, plant disease management, phytotoxicity

## Abstract

Multimodal antimicrobial technology is regarded as a promising strategy for controlling plant diseases because it enhances antimicrobial efficacy by blocking multiple pesticide-resistance pathways. In this work, a pH-responsive multimodal antimicrobial system was constructed based on ZIF-90 for the controlled release of kasugamycin (KSM). A series of physicochemical characterizations confirmed the successful fabrication of ZIF-90-KSM. The results indicated that the loading capacity of ZIF-90-KSM for KSM was approximately 6.7% and that the ZIF-90 nanocarriers could protect KSM against photodegradation effectively. The acid pH at the site of disease not only decompose the Schiff base bonds between KSM and ZIF-90, but also completely dissolved the nanocarriers. The simultaneous release of KSM and Zn^2+^ ions was able to achieve multimodal antimicrobial functions during disease occurs. A bioactivity survey indicated that ZIF-90-KSM had superior fungicidal activity and longer duration against *Magnaporthe oryzae* than KSM aqueous solution. In addition, the phytotoxicity assessment of ZIF-90-KSM on rice plants did not reveal any adverse effects. Therefore, ZIF-90-KSM prepared by Schiff base reaction has great potential for achieving synergistic antifungal functions and provides an eco-friendly approach to manage rice diseases.

## 1. Introduction

Pesticides, one of the effective methods to reduce the damage caused by pathogens, insects, and weeds, play a crucial role in improving crop productivity and quality [1]. However, only 0.1% of the active ingredients actually reach the target pests, and more than 90% of the applied conventional pesticide formulations are lost into the surrounding environment through runoff, migration spray drift, decomposition, and leaching [2,3,4]. The poor utilization of active ingredients results in frequent application of traditional pesticide formulations to achieve a satisfactory control effect [5,6]. The unreasonable and excessive use of pesticides not only leads to the resistance of pests and waste of resources but also causes the destruction of soil biodiversity and eutrophication of water [7,8]. Therefore, it is very urgent to develop a sustainable strategy for prolonging the effective duration, improving the utilization efficiency, and reducing the nontarget toxicity of pesticides.

In recent years, stimuli-responsive pesticide delivery systems based on nanocarriers have attracted widespread attention due to their ability to control pesticide release, improve target activity, and minimize the adverse effects on the environment [9,10,11,12]. Among various nanocarriers explored, Zn-based metal–organic frameworks (ZIF-90) as pesticide carriers for stimuli-responsive pesticide delivery systems have a number of unique advantages, such as facile synthesis route, high porosity, abundant aldehyde groups, outstanding biocompatibility, and being environmentally friendly [13,14,15,16]. In addition, the Zn^2+^ ions released from ZIF-90 have obvious antimicrobial properties against various pathogens (such as Gram-negative *Escherichia coli* and Gram-positive *Staphylococcus aureus*) [17,18,19]. More importantly, the highly reactive-free aldehyde groups of ZIF-90 can be covalently conjugated with the amino groups in drug molecules via a Schiff base reaction [20,21]. The resulting ZIF-90-based nanomaterials can be rapidly degraded under acidic conditions to simultaneously release Zn^2+^ ions and active ingredients on demand for synergistic antimicrobial effects [22].

As an aminoglycoside antibiotic isolated from *Streptomyces kasugaensis*, kasugamycin (KSM) has excellent antimicrobial activity against *Erwinia amylovora*, *Aciovorax citrulli*, *Didymella segeticola*, *Rhizoctonia solani*, *Cercospora janseana*, and *Magnaporthe oryzae* [23,24,25,26,27]. Application of KSM before disease occurrence is an effective method to avoid the outbreak of plant diseases in the field. However, KSM is sensitive to sunlight and ultraviolet light, which results in its low utilization efficacy and short duration [28]. To overcome this problem, KSM incorporation into stable and biocompatible nanoparticles can enhance photostability and prolong the effective duration of active ingredients [29]. Unfortunately, in situ capture or postsynthesis adsorption via porous nanomaterials can lead to the instability of pesticide controlled-release systems due to aspecific release during delivery, which limits its wide application in the field of agriculture [30].

Rice (*Oryza sativa* L.) is an important food crop that feeds more than 3.5 billion people worldwide [31]. However, as a recognized cancer of rice, rice blast disease caused by *Magnaporthe oryzae* has a serious adverse impact on rice production every year, resulting in an annual yield loss of 10–35%, and even over 50% during disease epidemics the disease [32]. Previous researches have reported that *Magnaporthe oryzae* can manipulate the pH of host cells by secreting organic acids to affect the virulence of pathogens [33,34]. Therefore, pH is an efficient stimulus response factor of pesticide controlled-release system, which can achieve rapid targeted release of active ingredients and control plant diseases in timely fashion. In this study, an acid pH-stimuli responsive pesticide delivery system was constructed by introducing Schiff base bonds between ZIF-90 and KSM. The acid environment caused by *Magnaporthe oryzae* infection triggered degradation of ZIF-90-KSM. The KSM and Zn^2+^ ions released from ZIF-90-KSM induced the death of *Magnaporthe oryzae*. The characterization, light stability, release profiles, fungicidal activities, and safety of the prepared ZIF-90-KSM were fully evaluated.

## 2. Experimental

### 2.1. Materials

Kasugamycin (KSM; purity 80%) was purchased from Hubei Jiufenglong Chemical Co., Ltd. (Wuhan, China). KSM aqueous solution (6%) was purchased from Shanxi Xinyuan Huakang Chemical Co., Ltd (Shanxi, China). Imidazole-2-carboxaldehyde (ICA), sodium dodecyl sulfate (SDS), zinc nitrate hexahydrate, cetyltrimethylammonium bromide (CTAB), polyvinylpyrrolidone K30 (PVP), tert-butanol, tris(hydroxymethyl) aminomethane (Tris), and hydrochloric acid (HCl) were purchased from Shanghai Aladdin Biochemical Technology Co., Ltd. (Shanghai, China). Citric acid, sodium citrate, sodium hydroxide, acetic acid, ethanol, and chromatographic grade methanol were acquired from Adamas Beta Co., Ltd. (Shanghai, China). Deionized water and ultrapure water were prepared by a Milli-Q water purification system (Millipore, Milford, MA, USA).

### 2.2. Synthesis of ZIF-90-KSM

#### 2.2.1. Synthesis of ZIF-90

The ZIF-90 was synthesized by a water–alcohol-based method [35]. Briefly, 371.25 mg of zinc nitrate hexahydrate and 4.0 mg of CTAB were dissolved in the mixture of 20 mL H_2_O/tert-butanol (1: 1, *v/v*) as a triggered solvent. Then, 480.0 mg of ICA and 50.0 mg of PVP were dissolved in the mixture of 20 mL H_2_O/tert-butanol (1: 1, *v/v*) as a modifier. After that, the triggered solvent was poured into the modifier and continuously stirred for 5 min at room temperature. Finally, a pale-yellow precipitate was formed. The ZIF-90 solid product was obtained by centrifugation (13,000 rpm, 10 min), washed with excess ethanol, and vacuum-dried at 60 °C for 12 h.

#### 2.2.2. Synthesis of KSM-linked ZIF-90 (ZIF-90-KSM)

The KSM was covalently linked to the ZIF-90 through Schiff base reaction [36]. Firstly, 100 mg of KSM was dissolved in 3 mL of Tris-HCl buffer (0.2 M, pH 8.0) at room temperature. Then, 50 mg of the prepared ZIF-90 was dispersed in 3 mL of Tris-HCl buffer (0.2 M, pH 8.0) by an ultrasonic cleaner. This solution was added to the flask containing KSM solution dropwise with constant stirring in the dark at room temperature for 24 h. Finally, the products were collected by centrifugation and washed with deionized water several times to remove unreacted KSM molecules. The precipitate was dried in a vacuum for further treatment.

### 2.3. Characterization

The morphologies of ZIF-90 and ZIF-90-KSM were acquired using a FEI Tecnai™ 12 transmission electron microscope (TEM) (Philips, Eindhoven, Netherlands) at an acceleration voltage of 120 kV. The crystalline phases were carried out by powder X-ray diffraction (XRD) performed on a Bruker D8 (Bruker Co. Ltd., Karlsruhe, Germany) Advance system using Cu Kα radiation (λ = 1.5418 Å). The chemical functional groups of ZIF-90 and ZIF-90-KSM were determined by a Nicolet iS20 Fourier-transform spectrophotometer (FTIR) (Thermo-Fisher, Waltham, USA) in transmission mode using the KBr pellet technique. The zeta potential of the samples was performed with a Nano-zs90 Nanosizer (Malvern Instruments, Malvern, UK). The content of KSM in ZIF-90-KSM was estimated by an SDT-Q600 thermogravimetric analyzer (TA Instruments-Waters LLC, New Castle, USA) under a nitrogen atmosphere with continuous heating from ambient temperature to 600 °C at a heating rate 10 °C/min. X-ray photoelectron spectroscopy (XPS) of the samples was performed on a Thermo ESCALAB 250Xi (Thermo-Fisher, Waltham, USA). The specific surface areas of the samples were determined by Brunauer–Emmett–Teller (BET) nitrogen adsorption at 77 K using ASAP 2020 analyzer (Micromeritics, Norcross, USA).

The concentration of KSM released from the samples was determined by a high-performance liquid chromatography (HPLC) system from Waters (Milford, MA, USA). The system consists of a Waters Alliance e2695 separations module equipped with a Waters 2487 dual λ absorbance detector and a 250 mm × 4.6 mm InertSustain C_18_ column (particle size 5 µm, GL Sciences Inc., Tokyo, Japan). The column compartment was kept at 35 °C. The mobile phase for KSM detection was consisted of 0.1% acetic acid and 0.1% SDS (aqueous phase A) in ultrapure water and chromatographic grade methanol (organic phase B). Separation was performed with an isocratic elution at a volume ratio of 40:60 (A:B). The flow rate was maintained at 1 mL/min and the injection volume was 20 μL. The ultraviolet detector for KSM was set at 210 nm. All mobile phases and samples used for HPLC analysis were filtered using a 0.45 μm membrane filter.

### 2.4. Controlled-Release Kinetics

The pesticide release behavior was investigated in citric acid–sodium citrate buffer solution with different pH values (pH 4.0, 5.5, and 7.0) by a dialysis method [37]. Briefly, 2 mL of ZIF-90-KSM dispersion (20 mg/mL) was transferred into a dialysis bag (molecular weight cutoff: 3500 Da). Then, the bag was submerged in 38 mL of citric acid–sodium citrate under slow magnetic stirring at room temperature. At predetermined time intervals, aliquots of the sample from the respective buffers were collected and the same volume of fresh citric acid–sodium citrate buffer solution was replenished. The content of KSM released from ZIF-90-KSM was analyzed by the HPLC system. All the release points for the experiments were measured in triplicate. The release behaviors of KSM from ZIF-90-KSM were evaluated by zero-order, first-order, Higuchi, Ritger–Peppas, and Hixson–Crow models.

(1) $$\text{Zero-order}\;\text{model}:\;\frac{M_{t}}{M_{z}}=kt$$

(2) $$\text{First-order}\;\text{model}:\;\frac{M_{t}}{M_{z}}=1-e^{-kt}$$

(3) $$\text{Higuchi}\;\text{model}:\;\frac{M_{t}}{M_{z}}=kt^{\frac{1}{2}}$$

(4) $$\text{Ritger–Peppas}\;\text{model}:\;\frac{M_{t}}{M_{z}}=kt^{n}$$

(5) $$\text{Hixson–Crowell}\;\text{model}:\;{\left(1-\frac{M_{t}}{M_{z}}\right)}^{\frac{1}{3}}=-kt$$

where *M_t_*/*M_z_* represents the amount of KSM released at time *t*; *k* is the kinetic constant that depends on incorporate characteristics of KSM and ZIF-90 system; and *n* is the diffusional exponent that characterizes the mechanism of KSM release.

### 2.5. Light Stability of ZIF-90-KSM

To evaluate the light stability of KSM, KSM aqueous solution and ZIF-90-KSM under ultraviolet light irradiation, the same concentration of samples was exposed to a 32 W ultraviolet lamp (254 nm) with a distance of 20 cm. Approximately 0.75 mL of the solution containing KSM from the respective samples was taken out at regular intervals, the residual amount of the samples was measured by the HPLC system. The degradation efficiency of KSM in the samples was calculated by the following equation:

(6) $$\text{Degradation}\;\text{efficiency}\;\left(\%\right)=\frac{C_{0}-C_{t}}{C_{0}}\times 100$$

The photodegradation of KSM in the samples was fitted using pseudo-first-order kinetics, as given by the following equation:

(7) $$\ln\frac{C_{t}}{C_{0}}=-kt$$

where *C_t_* is the concentration of KSM at irradiation time *t*, *C_0_* is the initial concentration of KSM, and *k* is the rate constant.

### 2.6. Antifungal Assays

The mycelium growth rate method was used to evaluate the antifungal activity of ZIF-90-KSM. A certain amount of ZIF-90-KSM was poured into molten potato dextrose agar (PDA, pH 5.7) medium, where all the concentrations were determined by the concentration of KSM. Then, 20 mL of the medium containing different concentrations of pesticide (1.5, 3.0, 6.0, 12.0, and 24.0 mg/L) was poured into each sterilized petri dish (9 cm in diameter). After solidification for 2 h, a mycelium plug (6 mm in diameter) taken from the 10-day-old culture of *Magnaporthe oryzae* was aseptically transferred to the center of the above PDA plates supplemented with ZIF-90-KSM. After culturing at 25 °C for 14 days in the dark, the colony diameter was measured three times in different perpendicular directions. To make a comparison, KSM aqueous solution and blank ZIF-90 were used as controls.

(8) $$\text{Mycelium}\;\text{growth}\;\text{inhibition}\;\text{rate}\;\left(\%\right)=\frac{D_{c}-D_{t}}{D_{c}}\times 100$$

where *D_c_* is the colony diameter of control at day 14, *D_t_* is the colony diameter of treatment at day 14.

### 2.7. Phytotoxicity of ZIF-90-KSM

Rice seeds (*Oryza sativa* L. cv Xiangliangyou 900) were surface sterilized with 75% ethanol (*v/v*) for 5 min, then treated with 5% sodium hypochlorite for 5 min and washed with deionized water several times. After soaking for 48 h in ultrapure water, the germinated seeds were transferred to plastic pots (9.2 cm × 9.2 cm × 8.5 cm) containing nutrient soil (15 plants per container). Plants were cultivated for two weeks in a growth chamber at a 26 °C/20 °C (day/night) cycle. The plants were treated with different concentrations of KSM aqueous solution and ZIF-90-KSM (0, 50, 100, and 200 mg/L), where all the concentrations were determined by the mass of KSM. Fourteen days after treatment, the shoot height, shoot fresh weight, shoot dry weight of rice, and the relative content of chlorophyll in plant leaves (expressed as SPAD index) were measured. All treatments were performed in triplicate.

### 2.8. Data Analysis

All statistical analyses were conducted using SPSS 23.0 statistical analysis software (SPSS, Chicago, IL, USA). The diameter of the nanomaterials was quantified by analyzing the TEM images using ImageJ software. The data were analyzed by Duncan multiple range test (*p* < 0.05) and presented as the mean ± standard error (SE). Origin 2022b (learning edition) was used to plot the graphs. All experiments were performed at least three times.

## 3. Results and Discussion

### 3.1. Preparation and Characterization of ZIF-90-KSM

**Figure 1:**
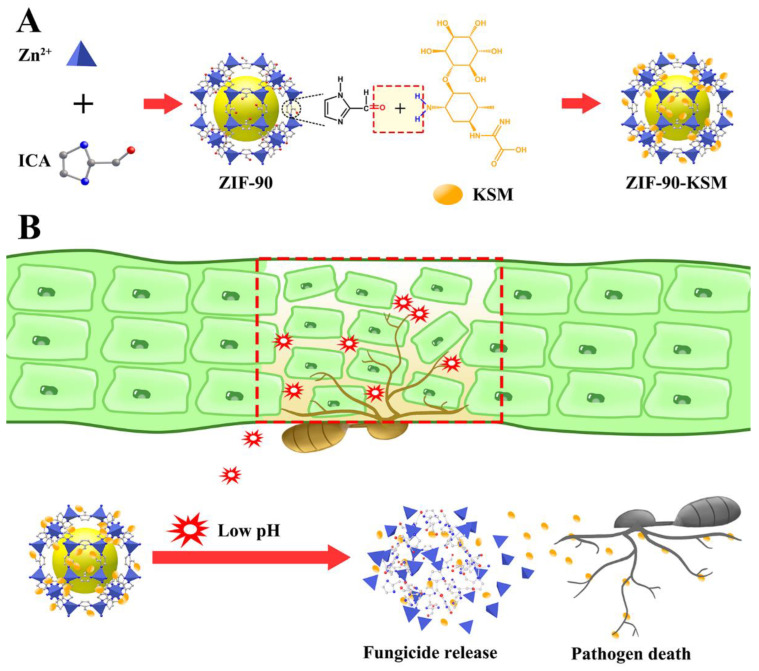
The mechanism for preparation of the ZIF-90-KSM (**A**). The triggered-release mechanism of KSM from ZIF-90-KSM (**B**).

Herein, ZIF-90-KSM with acid-triggered release properties was successfully constructed. The synthetic process and release mechanism of ZIF-90-KSM are summarized in Figure 1A. ZIF-90 was self-assembled from the linkage of ICA and Zn^2+^ ions in a water–alcohol-based system. Then, KSM was introduced into ZIF-90 via the Schiff base reaction of the aldehyde group in ICA and the amino group in KSM, forming acid-responsive ZIF-90-KSM.

**Figure 2:**
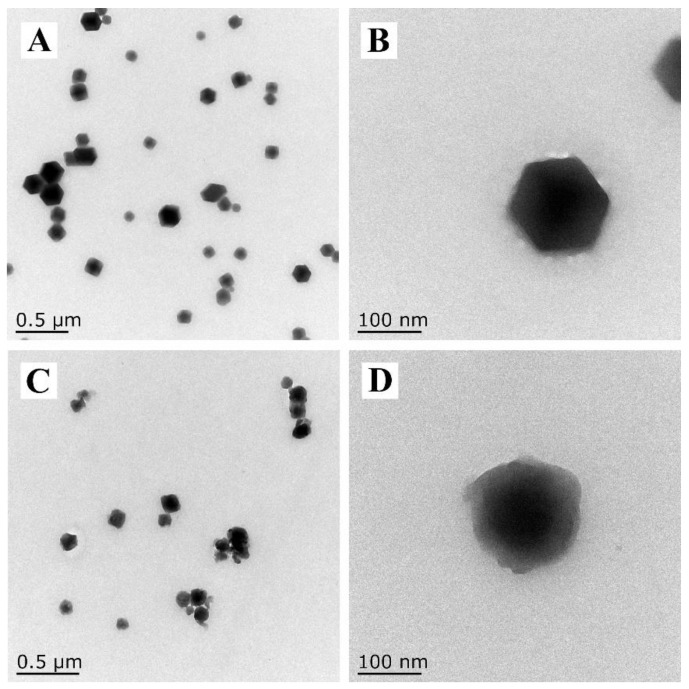
TEM images of ZIF-90 (**A**) ZIF-90-KSM (**C**). The images of (**B**,**D**) are higher magnification of TEM images of (**A**,**C**).

**Figure 3:**
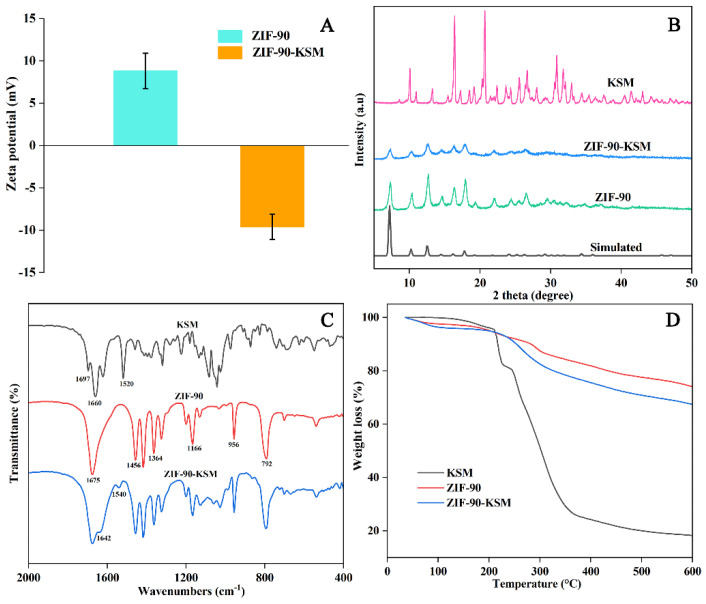
Zeta potential of ZIF-90 and ZIF-90-KSM (**A**), XRD patterns of KSM, ZIF-90 and ZIF-90-KSM (**B**), FTIR spectra of KSM, ZIF-90 and ZIF-90-KSM (**C**), and TGA curves of KSM, ZIF-90 and ZIF-90-KSM (**D**).

As depicted in Figure 2A,B, the synthesized ZIF-90 had good dispersibility and regular granatohedron structures with a diameter of around 100–200 nm. After KSM modification, the morphology of ZIF-90-KSM had a slight change compared to that of ZIF-90, and the original cubic structure became indistinct (Figure 2C,D). To qualitatively verify the formation processes of ZIF-90-KSM, the samples were analyzed by zeta potential measurement. As shown in Figure 3A, ZIF-90 had a zeta potential of 8.8 ± 2.1 mV derived from the positive charge of CATB, which was consistent with a previous report [38]. When the active ingredients were grafted on the ZIF-90, the zeta potential of ZIF-90-KSM shifted to −9.6 ± 1.5 mV. The reversed zeta potential attributed to the negative charge of KSM shielded the positively charged of the CTAB-capped ZIF-90 in the water.

The crystal structure changes of ZIF-90 after loading of KSM were monitored using XRD analysis. As shown in Figure 3B, the simulated XRD patterns of ZIF-90 exhibited six characteristic diffraction peaks at 5.03°, 8.36°, and 8.96°, which were assigned to the crystalline plane of (011), (200), (112), (022), (013), and (222), respectively [39,40]. According to the X-ray single crystal data, the characteristic peaks of the prepared ZIF-90 showed a good correlation with the simulated pattern of ZIF-90, indicating the successful coordination of the Zn^2+^ ions and ICA. After modification with KSM, the obtained ZIF-90-KSM had similar characteristic peaks but with lower intensity, indicating that KSM loading could destroy the framework integrity of ZIF-90. These findings were consistent with TEM observations of ZIF-90-KSM.

The compositions of KSM, ZIF-90, and ZIF-90-KSM were characterized by FTIR spectra (Figure 3C). For KSM, the peak at 1697 cm^−1^ was attributed to the C=O stretching vibration of the carbonyl group, while peaks at 1660 and 1520 cm^−1^ were assigned to the vibration of the amide I (N-H, stretching) and amide II (N-H, bending). The characteristic absorption bands of KSM are consistent with previous studies [41,42]. In the FTIR spectra for ZIF-90, the peak at 1675 cm^−1^ was attributed to C=O stretching vibration of the aldehyde groups in ICA ligand. Moreover, the peaks appeared at 1456 cm^−1^, 1364 cm^−1^, 1166 cm^−1^, 956 cm^−1^, and 792 cm^−1^ were belonged to the characteristic absorption of imidazole moieties in ZIF-90. After the reaction with KSM by Schiff base reaction, two new adsorption peaks assigned to the bending vibration of C=N and –NHCO– were observed at 1642 and 1540 cm^−1^, indicating the successful conjugation of KSM to ZIF-90. The results of FTIR spectra were consistent with the previous studies related to modifying ZIF-90 via Schiff base bonds [43,44].

The thermal stability of KSM, ZIF-90, and ZIF-90-KSM was investigated using thermogravimetric analysis (TGA). As shown in Figure 3D, the original weight loss in the temperature range of 35–223 °C that might be attributed to the removal of free and bound water molecules from the samples. The TGA curve of KSM reveals that the weight loss from 223 °C to 400 °C could be caused by the deformation of KSM hydrochloride [45]. In the case of ZIF-90, 26.0% weight loss at 223–600 °C is mainly due to the decomposition of the ICA linker present in ZIF-90. After functionalization with KSM, ZIF-90-KSM showed a more obvious weight loss (32.7%) in the temperature range of 223–600 °C, which could be due to the decomposition of the ZIF-90-KSM framework. From the results of TGA analyses, the KSM weight in ZIF-90-KSM was estimated to be 6.7%.

For further verification of the successful covalent-bonded immobilization of the KSM, the surface compositions were explored by XPS analyses. As shown in Figure S1A, the XPS survey spectra for ZIF-90 and ZIF-90-KSM confirmed the existence of Zn, O, N, and C elements. To further determine the binding states of O in ZIF-90 and ZIF-90-KSM, high-resolution XPS was used (Figure S1B,C). The O 1s spectrum of ZIF-90 was resolved into three distinct subpeaks at binding energy of 535.2 eV, 531.8 eV, and 531.1 eV, which were arisen from H_2_O, -CHO, and Zn-OH (Figure S1A) [46,47]. After the formation of ZIF-90-KSM, the peaks belonging to ZIF-90 disappeared and three new peaks attributed to C-OH, C-O, and C=O appeared at 533.2 eV, 532.4 eV, and 531.6 eV respectively, confirming that KSM successfully reacted with the aldehyde groups of ZIF-90 [48,49].

The surface area and porosity of the samples were investigated by nitrogen adsorption–desorption isotherms. As depicted in Figure S1D, the adsorption isotherm of ZIF-90 displayed typical type I and IV isotherm with an H4 hysteresis loop in the IUPAC classification, which indicates that there were a large number of micropores and mesopores inside it. The specific surface area was 553.26 m^2^/g, the pore volume was 0.27 cm^3^/g, and the average pore size was 2.00 nm. After modification with KSM, type IV adsorption isotherms with hysteresis loops at high pressure appeared, indicating that its micropores were greatly reduced relative to ZIF-90, with the mesopores and macropores dominating (96.60 m^2^/g for specific surface area, 0.06 cm^3^ g^−1^ for total pore volume, and 2.43 nm for average pore size) (Table S1). This was likely due to the loaded KSM blocking the pore structure of ZIF-90. According to the adsorption isotherm and pore size data in Table S1, the introduction of KSM might break the Zn-N coordination bonds in ZIF-90, leading to the collapse of the ZIF-90 structure [46]. As a result, the collapse of micropores reduced the specific surface area and increased the pore size.

### 3.2. Light Stability of ZIF-90-KSM

**Figure 4:**
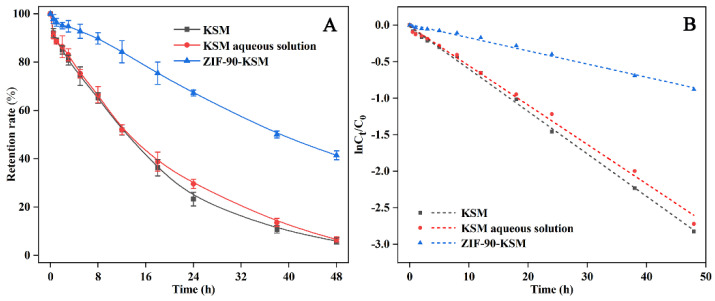
Stability of KSM, KSM aqueous solution and ZIF-90-KSM under ultraviolet radiation (**A**). Pseudo-first-order models of KSM photodegradation for KSM, KSM aqueous solution and ZIF-90-KSM (**B**).

As shown in Figure 4A, KSM aqueous solution and KSM dispersing in deionized water decomposed rapidly after being exposed to ultraviolet irradiation. After 48 h irradiation, the retention of KSM aqueous solution and KSM were only 6.58 ± 1.06% and 5.94 ± 1.30 respectively, which means that the aqueous solution had no protection for KSM. In contrast, after the KSM covalently cross-linked with the ZIF-90 via Schiff base reaction, the ZIF-90-KSM exhibited better light stability, and the retention of the active ingredients was 41.43 ± 1.88% after being exposed for 48 h. The degradation kinetics of the samples were well fitted with first-order decay equations ((ln *C_t_*/*C*_0_ = −*kt*) (Figure 4B and Table 1). According to the initial rate constant (*k*), the half-lives (*DT*_50_) of KSM, KSM aqueous solution, and ZIF-90-KSM were 11.92, 12.85, and 37.46 h, respectively. The overall comparison of various carriers used to protect active ingredients from photolysis is given in Table S2. For instance, KSM-loaded silica nanospheres exhibited a fourfold decrease in the photodegradation of the active ingredients compared with KSM after 72 h of UV irradiation. In this study, the half-life of ZIF-90-KSM was 3.1 times that of KSM. Compared with other reported carriers, ZIF-90 carriers could not only reduce the photolysis of the active ingredients, but also release Zn^2+^ ions to achieve a synergistic antimicrobial effect with KSM. Therefore, the ZIF-90-KSM provided a promising strategy for controlling plant diseases in modern agriculture.

### 3.3. Pesticide Loading and Controlled-Release Kinetics

The mechanism of KSM triggered release from ZIF-90-KSM is illustrated in Figure 1B. When the pathogens infect rice plants, the invasive hyphae of *Magnaporthe oryzae* could induce acidification of host cells, resulting in the cleavage of acid-sensitive Schiff base bonds, leading to the release of KSM on demand.

**Figure 5:**
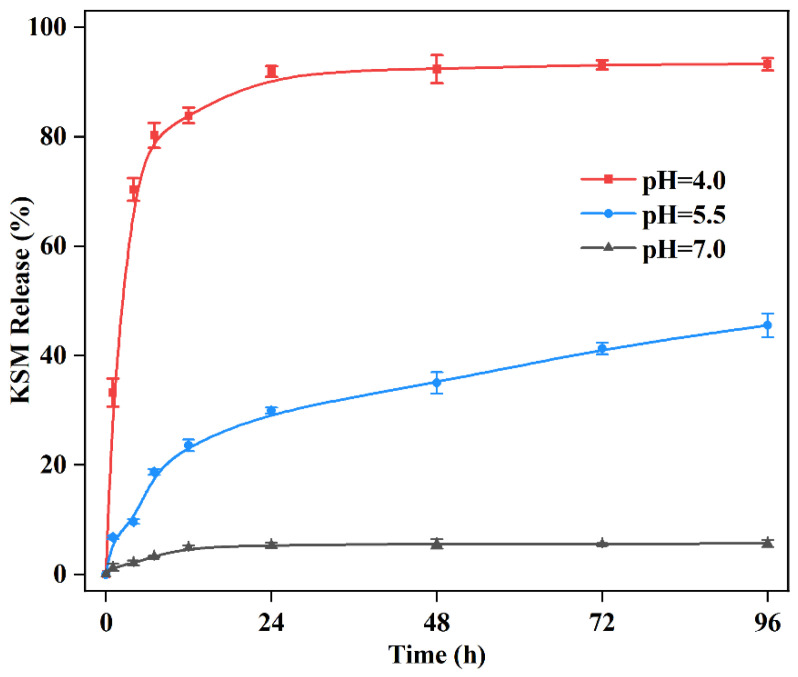
Effects of pH values on the release performances of KSM from ZIF-90-KSM.

Figure 5 shows the release profile KSM from ZIF-90-KSM at different pH values (4.0, 5.5, and 7.0). The ZIF-90-KSM displayed significantly pH-responsive properties, and the release of KSM molecules showed an increase in a typical pH-dependent manner. At pH 7.0, merely 5.67 ± 0.53% of KSM was released from ZIF-90-KSM after continuous incubation for 96 h, suggesting the excellent stability of the nanoparticles under neutral condition. By contrast, the released amount of KSM reached up to 45.53 ± 2.18% at pH 6.0, and 93.21 ± 1.39% at pH 4.5 within a period of 96 h, respectively. The faster release rate under acidic conditions was probably due to the fracture of the acid-responsive imine bond in ZIF-90-KSM, resulting in the release of KSM on-demand, which is advantageous for prolonging their effective duration.

To further investigate the release kinetics of KSM from ZIF-90, the data were fitted to five different kinetic models. The fitted parameter values were presented in Table 2. For the effects of pH values, the controlled-release kinetics of KSM best fitted with the Higuchi model. Thus, the release of KSM was governed by Fickian diffusion.

### 3.4. Fungicidal Activity

**Figure 6:**
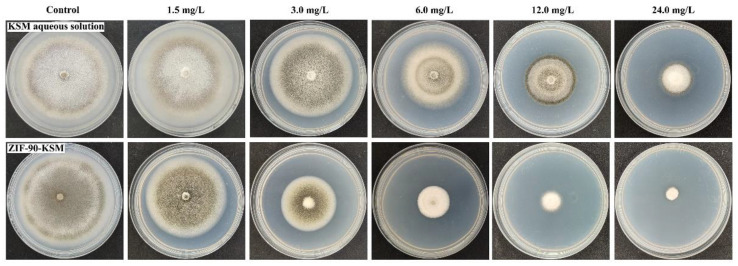
Images of the fungicidal activity of KSM aqueous solution and ZIF-90-KSM against *Magnaporthe oryzae*.

The fungicidal activities of ZIF-90, KSM aqueous solution, and ZIF-90-KSM against *Magnaporthe oryzae* were determined by the mycelium growth rate method. As shown in Figure 6, the growth inhibition rates of *Magnaporthe oryzae* colony were affected by KSM aqueous solution and ZIF-90-KSM in a typical dose-dependent manner. These results are in agreement with those reported by other researchers for nano-fungicides [25,50]. The toxicity regression equations, *EC*_50_ values, 95% confidence interval, and correlation coefficients (*r*) of the samples are listed in Table S3. The *EC*_50_ values of the KSM aqueous solution and ZIF-90-KSM were 4.54 and 2.33 mg/L, indicating that the fungicidal activity of ZIF-90-KSM was approximately two times higher than that of the KSM aqueous solution. Additionally, according to the results of mycelial growth diameter, the blank ZIF-90 vector also had antifungal activity at high concentration (Figure S2), which is mainly attributed to the antifungal effect of Zn^2+^ ions released from ZIF-90. The antimicrobial activities differences between ZIF-90-KSM and KSM aqueous solution can be explained as follows: the nanosized ZIF-90-KSM can facilitate the delivery at the target site, especially under the acidic conditions of pathogenic infection, the Schiff base bond cleavage facilitates the rapid release of KSM and zinc ions in ZIF-90-KSM, which can achieve a synergistic antifungal effect. Several studies have also reported that Zn^2+^ ions released from zinc-based metal–organic frameworks inhibited the growth of pathogens and achieve synergistic treatment with antibiotic fungicides [51,52,53]. Therefore, ZIF-90 would be a desirable carrier that can evidently enhance the antimicrobial activity of the loaded active ingredients.

### 3.5. Safety Assay

**Figure 7:**
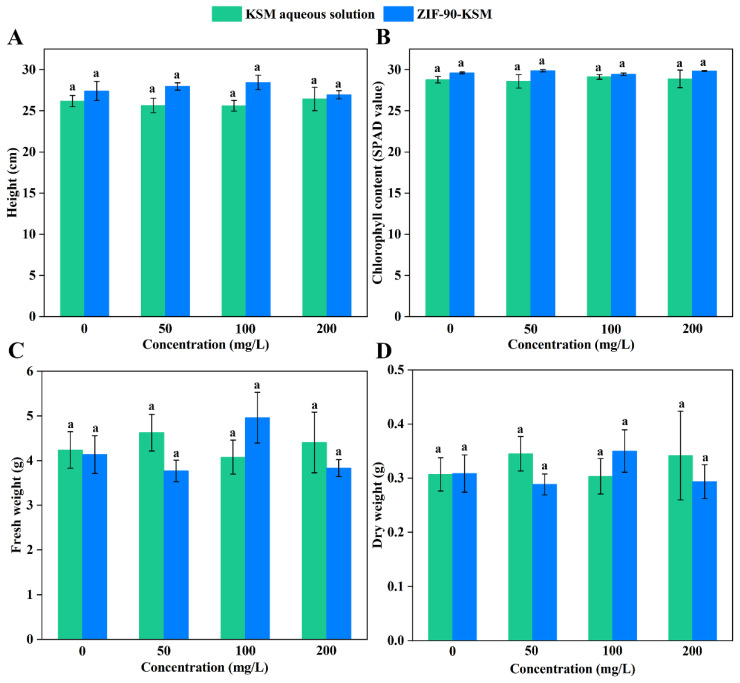
Influences of foliar application of KSM aqueous solution and ZIF-90-KSM on shoot height (**A**), leaf SPAD value (**B**), shoot fresh weight (**C**), and shoot dry weight (**D**) of rice plants. Different letters represent a statistically significant difference on Duncan’s multiple range test (*p* < 0.05).

Phenotypic observations of rice shoot height, shoot fresh weight, shoot dry weight, and leaf chlorophyll content (SPAD value) were used to evaluate the phytotoxicity of ZIF-90-KSM [54,55,56]. As shown in Figure 7A–D, the shoot height, shoot fresh weight, shoot dry weight, and leaf SPAD value of rice plants were not significantly affected by different concentrations of ZIF-90-KSM treatments compared to that of KSM aqueous solution treatments. Therefore, the ZIF-90-KSM prepared by Schiff base reaction has great potential for achieving synergistic antifungal functions and provides an eco-friendly approach to manage rice diseases.

## 4. Conclusions

In summary, ZIF-90-KSM was synthesized by conjugating ZIF-90 and KSM via an acid-cleavable Schiff base bond. The prepared ZIF-90-KSM had good dispersibility and regular granatohedron structures with a diameter of around 100–200 nm. The KSM loading capacity of ZIF-90-KSM was approximately 6.7%. The KSM conjugated with ZIF-90 improved the light stability of the active ingredients under sunlight. In addition, ZIF-90-KSM exhibited excellent pH stimuli-response performance, which synchronously released Zn^2+^ ions and KSM in acidic conditions. Bioactivity experiments showed that ZIF-90-KSM had strong fungicidal activity and long-term efficacy against *Magnaporthe oryzae*. More importantly, ZIF-90-KSM showed satisfactory safety during the growth of rice plants. Thus, the pH-responsive ZIF-90-KSM provides a new strategy for management of plant diseases and has bright prospects for controlling the release of pesticides.

## Figures and Tables

**Table 1:** Modeling parameters for photodegradation of KSM, KSM aqueous solution and ZIF-90-KSM under ultraviolet radiation.

Parameter	Pseudo-First-Order Kinetics		
	KSM	KSM Aqueous Solution	ZIF-90-KSM
*k* (h^−1^)	0.058	0.054	0.018
*Relative index* (*r*^2^)	0.998	0.995	0.992
*DT*_50_ (h) ^a^	11.92	12.85	37.46

^a^*DT*_50_ = dissipation half-life of KSM.

**Table 2:** Determined parameters of KSM released from ZIF-90-KSM by fitting several kinetic equations.

Condition	Kinetic Model	*k*(×10^−2^)	*n*	*r* ^2^
pH 4.0	Zero-order	1.38	—	0.6204
	First-order	3.85	—	0.7420
	Higuchi	13.15	—	0.8469
	Ritger–Peppas	45.43	0.19	0.7334
	Hixson–Crowell	0.19	—	0.3548
pH 5.5	Zero-order	0.58	—	0.8568
	First-order	0.75	—	0.8963
	Higuchi	5.09	—	0.9836
	Ritger-Peppas	6.86	0.43	0.9578
	Hixson-Crowell	0.14	—	0.8231
pH 7.0	Zero-order	0.08	—	0.7164
	First-order	0.08	—	0.7188
	Higuchi	0.75	—	0.9136
	Ritger-Peppas	1.49	0.33	0.8689
	Hixson-Crowell	0.02	—	0.5381

## Data Availability

Not applicable.

## Supplementary Materials

The following supporting information can be downloaded at: https://www.mdpi.com/article/10.3390/nano12203622/s1, Figure S1: Broad range XPS spectra of ZIF-90 and ZIF-90- KSM (A). Deconvolution of the high-resolution scan of O1s spectra of ZIF-90 (B) and ZIF-90-KSM (C). N_2_ adsorption–desorption isotherms of ZIF-90 and ZIF-90-KSM (D). Figure S2: Images of the fungicidal activity of ZIF-90 against *Magnaporthe oryzae*. Table S1: Surface and porosity properties of the samples. Table S2: Comparison of various carriers used to protect KSM from photolysis. Table S3: Fungicidal activity of KSM formulations as KSM aqueous solution and ZIF-90-KSM against *Magnaporthe oryzae*. References [29,41,42] were cited in the Supplementary Materials.
