# Supplementary material for: Zeolitic Imidazole Framework-90-Based Pesticide Smart-Delivery System with Enhanced Antimicrobial Performance
Source: Nanomaterials (Basel). 2022 Oct 15;12(20):3622. doi: 10.3390/nano12203622 (PMC9607848; doi:10.3390/nano12203622)
Supplement: Supplementary file 1 [file nanomaterials-12-03622-s001.zip › nanomaterials-1943514-supplementary.pdf]

Supplementary materials

Zeolitic Imidazole Framework-90-Based Pesticide Smart-Delivery System with Enhanced Antimicrobial Performance

You Liang [1], Sijin Wang [1], Hongqiang Dong [2], Siwen Yu [1], Huijuan Jia [1], Jin Wang [1], Yijia Yao [1], Yuanfeng Wang [1], Jiehui Song [1] and Zhongyang Huo [1*]

[1] Co-Innovation Center for Modern Production Technology of Grain Crop/Jiangsu Key Laboratory of Crop Genetics and Physiology, Yangzhou University, Yangzhou 225009, China

[2] Xinjiang ProductionandConstruction Corps Key Laboratory of Protection and Utilization of Biological Resources in Tarim Basin, Tarim University, Alaer 843300, China

* Correspondence: huozy69@163.com; Tel.: +86-514-87972363; Fax: +86-514-87972363

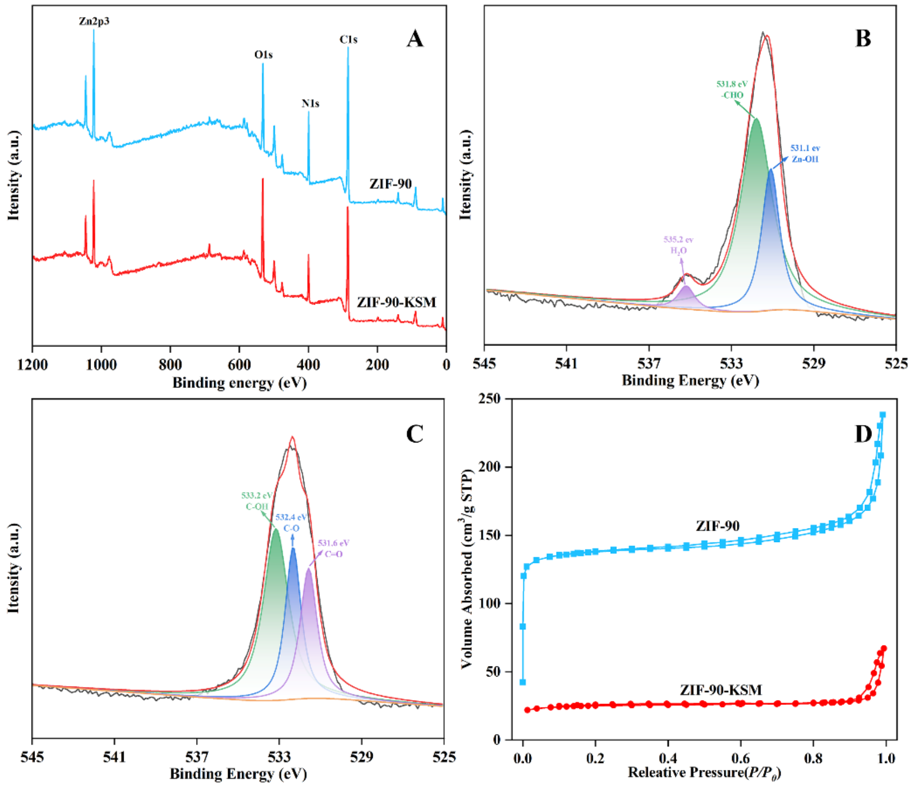

Figure S1. Broad range XPS spectra of ZIF-90 and ZIF-90- KSM (A). Deconvolution of the high-resolution scan of O1s spectra of ZIF-90 (B) and ZIF-90-KSM (C). N_2 adsorption–desorption isotherms of ZIF-90 and ZIF-90-KSM (D)

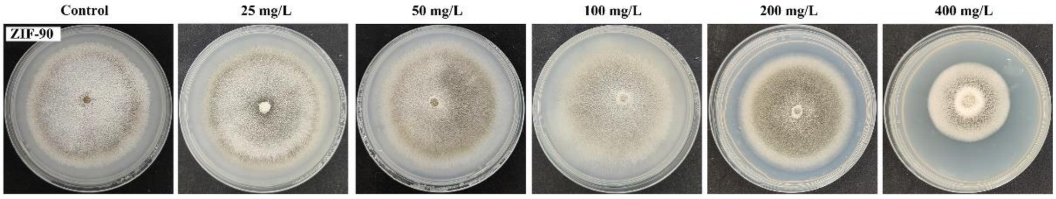

Figure S2. Images of the fungicidal activity of ZIF-90 against *Magnaporthe oryzae*

Table S1. Surface and porosity properties of the samples.

Sample	BET surface area (m^2/g)	Pore volume (cm^3/g)	Average pore size (nm)
ZIF-90	553.26	0.27	2.00
ZIF-90-KSM	96.60	0.06	2.43

Table S2. Comparison of various carriers used to protect KSM from photolysis.

Carrier	UV-light stability			Ref.
	UV Light	Sample state	Degradation rate	
Pectin-conjugated silica microcapsules	light intensity, 700 μW cm^{-2}	Suspension in PBS solution	The degradation is 5 times lower than that of technical after 60 h	[29]
Pectin	light intensity, 700 μW cm^{-2}	Suspension in PBS solution	Reduced degradation by approximately 30%	[41]
Silica nanospheres	light intensity, 700 μW cm^{-2}	Suspension in PBS solution	The degradation is 4 times lower than that of technical after 72 h	[42]

Table S3. Fungicidal activity of KSM formulations as KSM aqueous solution and ZIF-90-KSM against *Magnaporthe oryzae*.

Treatment	Regression equation	EC_{50} (95% confidence interval) (mg/L)	Correlation coefficient (r)
KSM aqueous solution	y=0.79+1.20x	4.54 (3.95-5.17)	0.987
ZIF-90-KSM	y=0.65+1.76x	2.33 (2.04-2.61)	0.993

Notes: EC_{50} Inhibitory concentration that inhibit 50% of the exposed fungus.

[29] Fan, C.; Guo, M.; Liang, Y.; Dong, H.; Ding, G.; Zhang, W.; Tang, G.; Yang, J.; Kong, D.; Cao, Y. Pectin-conjugated silica microcapsules as dual-responsive carriers for increasing the stability and antimicrobial efficacy of kasugamycin. *Carbohydr. Polym.* **2017**, *172*, 322–331. https://doi.org/10.1016/j.carbpol.2017.05.050.

[41] Liu, Y.; Sun, Y.; Ding, G.; Geng, Q.; Zhu, J.; Guo, M.; Duan, Y.; Wang, B.; Cao, Y. Synthesis, characterization, and application of microbe-triggered controlled-release kasugamycin–pectin conjugate. *J. Agric. Food Chem.* **2015**, *63*, 4263–4268. https://doi.org/10.1021/jf5055062.

[42] Ding, G.; Li, D.; Liu, Y.; Guo, M.; Duan, Y.; Li, J.; Cao, Y. Preparation and characterization of kasuga-silica-conjugated nanospheres for sustained antimicrobial activity. *J. Nanopart. Res.* **2014**, *16*, 1–10. https://doi.org/10.1007/s11051-014-2671-0.
